# Highland deer mice support increased thermogenesis in response to chronic cold hypoxia by shifting uptake of circulating fatty acids from muscles to brown adipose tissue

**DOI:** 10.1242/jeb.247340

**Published:** 2024-04-11

**Authors:** Sulayman A. Lyons, Grant B. McClelland

**Affiliations:** Department of Biology, McMaster University, Hamilton, ON, Canada, L8S 4K1

**Keywords:** [1-^14^C]2-Bromopalmitic acid, Non-esterified fatty acids, *Peromyscus maniculatus*, Skeletal muscle, Heat production, Phenotypic plasticity

## Abstract

During maximal cold challenge (cold-induced *V̇*_O___2_,max_) in hypoxia, highland deer mice (*Peromyscus maniculatus*) show higher rates of circulatory fatty acid delivery compared with lowland deer mice. Fatty acid delivery also increases with acclimation to cold hypoxia (CH) and probably plays a major role in supporting the high rates of thermogenesis observed in highland deer mice. However, it is unknown which tissues take up these fatty acids and their relative contribution to thermogenesis. The goal of this study was to determine the uptake of circulating fatty acids into 24 different tissues during hypoxic cold-induced *V̇*_O___2_,max_, by using [1-^14^C]2-bromopalmitic acid. To uncover evolved and environment-induced changes in fatty acid uptake, we compared lab-born and -raised highland and lowland deer mice, acclimated to either thermoneutral (30°C, 21 kPa O_2_) or CH (5°C, 12 kPa O_2_) conditions. During hypoxic cold-induced *V̇*_O___2_,max_, CH-acclimated highlanders decreased muscle fatty acid uptake and increased uptake into brown adipose tissue (BAT) relative to thermoneutral highlanders, a response that was absent in lowlanders. CH acclimation was also associated with increased activities of enzymes citrate synthase and β-hydroxyacyl-CoA dehydrogenase in the BAT of highlanders, and higher levels of fatty acid translocase CD36 (FAT/CD36) in both populations. This is the first study to show that cold-induced fatty acid uptake is distributed across a wide range of tissues. Highland deer mice show plasticity in this fatty acid distribution in response to chronic cold hypoxia, and combined with higher rates of tissue delivery, this contributes to their survival in the cold high alpine environment.

## Teaser

**Highlighted Article:** High-altitude native deer mice increase brown adipose tissue fatty acid uptake to support maximal heat production following acclimation to cold hypoxic conditions.

## INTRODUCTION

Lipids are known to be the primary substrate used in humans and other mammals to support low intensity activities, such as moderate aerobic exercise (Schippers et al., 2014). Lipids are also the primary substrate used for thermogenesis in rodents, at both submaximal and peak rates of heat production (Vaillancourt et al., 2009; Lyons et al., 2021). The primary consumer of oxygen and substrates during exercise are the skeletal muscles (Armstrong et al., 1992), and as work intensities increase, their recruitment pattern tracks changes in substrate use from primarily lipids to exclusively carbohydrates, at least for humans and the other mammals studied (Laughlin and Armstrong, 1982; Schippers et al., 2014). The situation is different during thermogenesis, as small eutherian mammals recruit both brown adipose tissue (BAT) and skeletal muscle for non-shivering thermogenesis (NST) and the shivering form of thermogenesis. These thermo-effector tissues contribute to maximal cold-induced rates of oxygen consumption (*V̇*_O___2_,max_), supported by lipids as the principal substrate (Lyons et al., 2021). In rodents, it is unclear which of the skeletal muscles participate in shivering and how substrates are partitioned between muscles, BAT, and other tissues at high rates of thermogenesis. It is also unknown if variation in substrate uptake by the thermo-effector tissues: (1) occurs between animals living in different environments, and/or (2) is a target of environmentally induced plasticity.

Deer mice (*Peromyscus maniculatus* Wagner 1845) native to high-altitude environments have evolved a variety of mechanisms to survive persistent low temperatures and low partial pressures of oxygen. For example, highland native deer mice are capable of elevated rates of lipid oxidation to support high rates of heat production in hypoxia (Lyons et al., 2021). These elevated rates of lipid oxidation in highlanders have been attributed to higher rates of NST and a greater capacity for circulatory lipid transport compared with lowlanders (Coulson et al., 2021; Lyons and McClelland, 2022). Indeed, circulatory delivery rates of free fatty acids were found to be approximately 3-fold greater in highland versus lowland deer mice during cold-induced *V̇*_O___2_,max_ in hypoxia (Lyons and McClelland, 2022).

During thermogenesis, it is unclear how lipid uptake is distributed among the skeletal muscles and BAT, especially in small endotherms, such as deer mice. Previous work in lab rats and mice have shown that acute cold exposure increases blood flow to thermo-effector tissues (Foster and Frydman, 1979) and enhances triglyceride-rich lipoprotein uptake in BAT (Bartelt et al., 2011). Furthermore, lab rats demonstrate a shunting of blood flow away from skeletal muscle to be redirected towards BAT following cold acclimation (Foster and Frydman, 1979). An increase in blood flow to the thermo-effector tissues during cold exposure would highlight their increased metabolic activity and need for uptake of circulating fatty acids to support heat production. However, this response may vary in rodents native to different thermal environments, or with differences in oxygen availability, such as populations of deer mice living at different altitudes.

Any differences in circulatory fatty acid uptake between lowland and highland deer mice would reflect altitude ancestry and/or evolved differences in phenotypic plasticity. Indeed, highland deer mice demonstrate unique acclimation responses to simulated high-altitude conditions by increasing rates of circulatory fat delivery, while lowland mice do not (Lyons and McClelland, 2022). Additionally, in response to cold hypoxia (CH) acclimation, highland deer mice increase NST, increase expression of uncoupling protein 1 (UCP-1) in BAT and increase UCP-1-stimulated respiration, whereas lowlanders fail to demonstrate a significant acclimation response (Coulson et al., 2021). Indeed, hypoxia may antagonize cold-induced phenotypic plasticity in lowland native rodents (Beaudry and McClelland, 2010). Given these population-specific responses in lipid oxidation and NST with acclimation to high-altitude conditions, we hypothesized that highland deer mice will show a unique acclimation response to cold hypoxia by increasing fatty acid uptake into BAT, whereas lowland deer mice will not.

To test this hypothesis, we assessed circulatory free fatty acid tissue uptake using [1-^14^C]2-bromopalmitic acid across 24 individual tissues, including central organs (liver, heart and diaphragm), white adipose tissue depots (inguinal and gonadal), BAT depots (interscapular and auxiliary) and 16 individual skeletal muscles of the forelimb, hindlimb and trunk. Quantifying fatty acid uptake across tissues reflects blood flow and metabolic rate, tissue-specific lipid metabolism, and provides a comprehensive picture of thermo-effector recruitment. We also examined the role of environmentally induced plasticity in tissue fatty acid uptake by acclimating deer mice from both populations to either thermoneutral normoxic or CH conditions. In some tissues, fatty acid uptake was compared with the protein abundance of membrane transporters and activities of key metabolic enzymes.

## MATERIALS AND METHODS

### Common garden experiment

All procedures were approved by the McMaster University Animal Research Ethics Board in accordance with guidelines set by the Canadian Council on Animal Care. Mice used in this study were part of an established breeding colony of highland (*P.m. rufinus*) and lowland (*P.m. nebracensis*) deer mice at McMaster University as previously described (Lui et al., 2015). Briefly, wild highland deer mice were trapped in Mount Blue Sky, CO (4350 m a.s.l.) and lowland deer mice were trapped at a similar latitude in Kearney, NE, USA (656 m a.s.l.). Wild mice were transferred to McMaster University (90 m a.s.l.) and housed in common laboratory conditions at ∼23°C, on a 12 h:12 h light:dark cycle, with unlimited access to food (Teklad Global 18% Protein Rodent Diet) and water. Mice were bred within their respective populations to produce second generation laboratory-born and -raised mice. Mice in this study were at least 6 months of age, and a mix of both males and females. Highland and lowland deer mice were randomly assigned to one of two acclimation groups, thermoneutral (TN, 30°C and 21 kPa O_2_) using a temperature controlled rodent incubator (Powers Scientific Inc., Doylestown, PA, USA) or cold hypoxia (CH, 5°C and 12 kPa O_2_, using hypobaric chambers; McClelland et al., 1998; Lyons et al., 2021) in a climate-controlled room, simulating high altitude (∼4300 m). Deer mice acclimated to CH were first housed at 5°C for 24 h at normobaria before being placed in hypobaric chambers. For routine cage cleaning and replenishment of food and water, CH deer mice were returned to normobaria for less than 30 min once per week. All mice were kept in their respective acclimation conditions for 6–8 weeks.

### ^14^C-Bromopalmitic acid infusate preparation

To trace the fate of circulating fatty acid during *V̇*_O___2_,max_, 1-^14^C labelled 2-bromopalmitic acid was used. 2-Bromopalmitic acid is transported and taken up by tissues using the same mechanisms as palmitic acid; however, once this 2-bromopalmitic fatty acid enters the cell, the 2-bromo group prevents its mitochondrial oxidation, trapping the fatty acid in the cytosol (Oakes et al., 1999). Preparation of bromopalmitic acid infusate was modified from previous methods (Oakes et al., 1999; Schönke et al., 2018). On the day of the experiment, 10^7^ dpm [1-^14^C]2-bromopalmitic acid organic stock solution (0.0045 mCi, or 45 μl bromopalmitic acid stock, MC 451, Moravek Inc., CA) was dried under N_2_ at room temperature in a glass test tube and reconstituted in 100 μl unlabeled infusate (saline containing 1.2% bovine serum albumin, Millipore Sigma, St Louis, MO, USA, and 0.15 mmol l^−1^ palmitic acid, Sigma Aldrich). The labelled infusate was then gently mixed using a 37°C water bath for 1 h.

### ^14^C-Bromopalmitic acid injection and maximal cold challenge

To facilitate tail vein injection, tails of the deer mice were cleaned of hair 48 h prior to trial. Mice were weighed and then were placed in a tail veiner restraint (Braintree Scientific, Inc., Braintree, MA, USA). Using a 27-gauge needle, 100 μl ^14^C-bromopalmitate infusate (4.5 µCi) was injected via the tail vein. Mice were then immediately exposed to maximal cold challenge conditions for 12 min to determine cold-induced *V̇*_O___2_,max_, as described in Lyons et al. (2021). In brief, hypoxic cold-induced *V̇*_O___2_,max_ was determined by pushing heliox (12% O_2_, 88% He) at 1000 ml min^−1^ through copper coils housed inside a temperature control cabinet and into a respirometry chamber (volume ∼500 ml) cooled to −10°C, using mass flow meters and controllers (Sierra Instruments, Monterey, CA, USA; MFC-4, Sable Systems, NV). Cold-induced *V̇*_O___2_,max_ was determined as the highest 10 s of *V̇*_O_2__ over the course of the entire trial. Similarly to our previous work (Lyons et al., 2021), whole-animal lipid oxidation rates were calculated using the indirect calorimetry equations from Frayn (1983). After 12 min in these conditions, mice were anesthetized using an isoflurane-soaked cotton ball. At exactly 15 min after the time of injection, mice were decapitated, blood and tissue were collected, flash frozen in liquid N_2_, and stored at −80°C until future processing. Tissues were dissected in the following order for all mice: soleus, red gastrocnemius, white gastrocnemius, tibialis anterior, extensor digitorum longus, rectus femoris, vastus lateralis, vastus medialis, semitendinosus, biceps femoris, gluteus, interscapular brown adipose tissue, erector spinae, trapezius, auxiliary brown adipose tissue, biceps, triceps, masseter, inguinal white adipose tissue, gonadal white adipose tissue, liver, diaphragm, right ventricle and left ventricle.

### Tissue processing and analysis

The processing of tissues for ^14^C-activity was modified from methods used by Schönke et al. (2018). Frozen tissues were weighed and placed in a vial containing 1 mol l^−1^ NaOH (30 µl mg^−1^ tissue), and briefly minced. The vial was then sealed and placed in a rotating water bath at 50°C for 1.5 h. After the tissue dissolved, an equivalent volume of 1 mol l^−1^ HCl was added to the vial to neutralize the solution. Then, 200 µl of the dissolved tissue was placed in a glass scintillation vial containing 2 ml scintillation fluid (Ecoscint A, National Diagnostics, Atlanta, GA, USA). Counts per minute (CPM) of ^14^C were measured for 5 min using a Tricarb 2900 TR liquid scintillation analyzer using QuantaSmart 1.31 (Packard Instrument) analysis software.

### Enzyme apparent *V*_max_

We measured the apparent maximal activities (*V*_max_) of β-hydroxyacyl-CoA dehydrogenase (HOAD), citrate synthase (CS) and cytochrome *c* oxidase (COX) in the red gastrocnemius, white gastrocnemius, tibialis anterior, rectus femoris, erector spinae and interscapular brown adipose tissue using a SpectraMax Plus 384 plate reader (Molecular Devices, Sunnyvale, CA, USA), as described previously for deer mice (Lyons and McClelland, 2022). Activities of HOAD and COX were measured on fresh homogenates, whereas CS activity was measured after homogenates had been frozen and thawed twice. Approximately 30 mg of powdered tissue was homogenized on ice using a glass-on-glass homogenizer in buffer (20 µl mg^−1^ tissue) containing 100 mmol l^−1^ potassium phosphate (pH 7.2), 5 mmol l^−1^ EDTA and 0.1% Triton X-100. Assays were performed at 37°C in triplicate, and controls for background activities were determined for each assay by omitting substrate. Assay conditions were the following: COX: 0.1 mmol l^−1^ of reduced cytochrome *c* (omitted in control) in 100 mmol l^−1^ K_2_HPO_4_ (pH 7.0) at an absorbance of 550 nm. HOAD: 0.1 mmol l^−1^ acetoacetyl-CoA (omitted in control), and 0.28 mmol l^−1^ NADH in 100 mmol l^−1^ triethanolamine·HCl (pH 7.0) at an absorbance of 340 nm. CS: 0.5 mmol l^−1^ oxaloacetate (omitted in control), 0.22 mmol l^−1^ acetyl-CoA and 0.1 mmol l^−1^ dithiobisnitrobenzoic acid (DTNB) in 40 mmol l^−1^ Tris (pH 8.0) at an absorbance of 412 nm.

### Western blotting for FAT/CD36

Fatty acid translocase CD36 (FAT/CD36) protein abundance in whole tissue homogenates of interscapular BAT (iBAT), erector spinae, red gastrocnemius and white gastrocnemius was determined using western blot analysis as previously described (Lyons and McClelland, 2022). Powdered tissue samples were homogenized using a motorized homogenizer in 25 volumes of ice-cold RIPA buffer (150 mmol l^−1^ NaCl, 50 mmol l^−1^ Tris·HCl, 1.0% Triton X-100, 0.5% deoxycholic acid, and 0.1% SDS, at pH 8.0). Total homogenate protein concentrations were quantified using Bradford assay (Bio-Rad Laboratories Ltd, Mississauga, ON, Canada). The protein isolates were diluted 1:1 in 2× Laemmli buffer [65.8 mmol l^−1^ Tris-HCl, pH 6.8, 2.1% SDS, 26.3% (w/v) glycerol, 0.01% Bromophenol Blue, and 10% β-mercaptoethanol] and were denatured for 5 min at 95°C. Denatured protein (20 μg for BAT and 40 μg for muscles) were separated on precast 12% sodium dodecyl sulfate-polyacrylamide gels (Bio-Rad) for 30 min at 100 V and then 45 min at 150 V in a Mini-Protein Tetra System (Bio-Rad). Separated proteins were then transferred to polyvinylidene difluoride membranes (Bio-Rad) using the Transblot Turbo Transfer System (Bio-Rad) at 25 V for 7 min. Membranes were incubated overnight at 4°C with blocking buffer (5% skimmed milk in 1× phosphate buffered saline, 0.1% Tween). The following day, membranes were incubated with primary antibody against FAT/CD36 (CD36 polyclonal antibody, Invitrogen, PA-16813) at a dilution of 1:500 in blocking buffer for 1 h at room temperature, followed by an incubation with HRP-conjugated secondary antibody (goat anti-rabbit, Invitrogen, cat. #31466) at dilution of 1:5000 in blocking buffer for 1 h at room temperature. Band densities were detected by chemiluminescence and normalized to total lane protein determined using Coomassie Blue (Bio-Safe™ Coomassie Stain #1610786, Bio-Rad). Images were taken and analyzed using a ChemiDoc MP Imaging System (Bio-Rad) and the Image Lab software package (Bio-Rad), respectively (Lui et al., 2015; Lyons and McClelland, 2022).

### Statistical analysis

We employed a 2-way analysis of variance (ANOVA), using body mass as covariate, to assess the main effects of deer mouse population, acclimation condition and their interaction. Pairwise Holm–Šídák *post hoc* tests were performed to evaluate significant interactions and we set the value for statistical significance to *P*<0.05. While sex was initially included as a random variable in our statistical model, we found no significant effect of sex for *V̇*_O___2_,max_, respiratory exchange ratio or whole-animal lipid oxidation, consistent with previous work (Lyons et al., 2021). Therefore, we grouped males and females for the analysis of population and acclimation differences. Statistical analyses were performed using the lme4 package (https://CRAN.R-project.org/package=lme4) in R v.4.2.0 (https://www.r-project.org) and Prism software (version 5.01; GraphPad Software, San Diego, CA, USA). All data are presented as means±s.e.m.

## RESULTS

### Respiration at thermogenic capacity

**Fig. 1.**
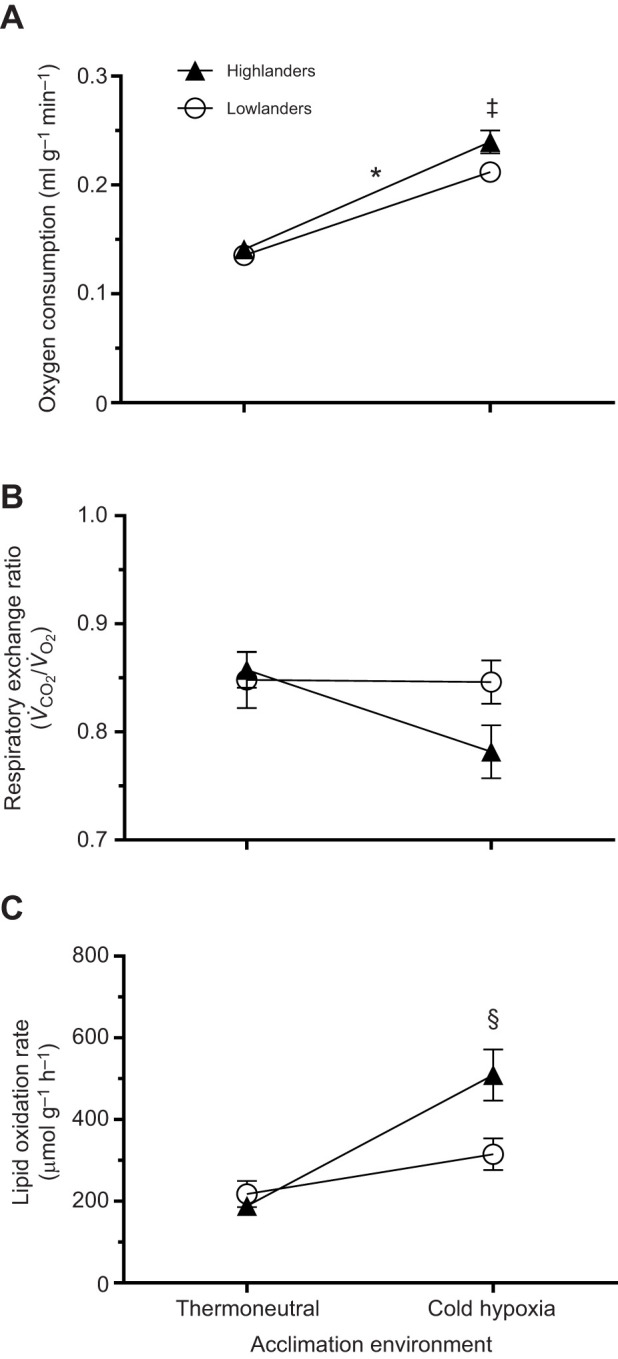
**The thermogenic capacity of second-generation laboratory-born and -raised highland and lowland deer mice (*Peromyscus maniculatus*).** (A) Hypoxic cold-induced maximal oxygen consumption (*V̇*_O___2_,max_, in ml g^−1^ min^−1^), (B) respiratory exchange ratios (RER= *V̇*_CO_2__/*V̇*_O_2__) and (C) whole-animal lipid oxidation rates (µmol g^−1^ h^−1^) in deer mice acclimated to thermoneutral (TN; 30°C, 21 kPa O_2_) or cold hypoxia (CH; 5°C, 12 kPa O_2_) conditions. *Significant difference between populations (main effect; *P*<0.05). ^‡^Significant difference between acclimations (main effect; *P*<0.05). ^§^CH highlanders are significantly different from TN highlanders (*post hoc* test; *P*<0.05). Sample sizes for TN and CH were *N*=24 and *N*=20 for highlanders, and *N*=25 and *N*=22 for lowlanders, respectively. Data are presented as means±s.e.m.

Hypoxic cold-induced *V̇*_O___2_,max_ was used to assess thermogenic capacity in lowland and highland deer mice acclimated to TN or CH conditions. Overall, we found that thermogenic capacity was 4–13% greater in highland deer mice compared with lowlanders (Fig. 1A; significant effect of population, *F*_1,87_=5.49; *P*=0.02), whereas CH acclimation led to an increase in *V̇*_O___2_,max_ of roughly 1.6-fold (significant effect of acclimation, *F*_1,87_=153.0; *P*<0.0001). To help understand the possible population differences in substrate use at *V̇*_O___2_,max_ and the influence of CH acclimation, we calculated the respiratory exchange ratios (RERs). We found that RER at *V̇*_O___2_,max_ ranged from 0.75 to 0.85, but there were no significant differences between populations (*F*_1,85_=1.52; *P*=0.22) or between acclimations (*F*_1,85_=2.99; *P*=0.09). These findings suggest that lipids are the main metabolic substrate for powering maximal thermogenesis (Fig. 1B). Interestingly, whole-animal lipid oxidation rates showed a significant population×acclimation interaction (*F*_1,85_=7.79; *P*=0.007), where only highlanders increased lipid oxidation rates (by 2.7-fold) following CH acclimation (*P*<0.05) (Fig. 1C). Furthermore, highlanders had lipid oxidation rates that were approximately 1.6-fold greater than lowlanders after CH acclimation (*P*<0.05). In contrast, TN-acclimated lowlanders and highlanders showed no significant difference in rates of lipid oxidation at hypoxic cold-induced *V̇*_O___2_,max_ (*P*>0.05).

### Fatty acid tissue uptake during thermogenesis

**Fig. 2.**
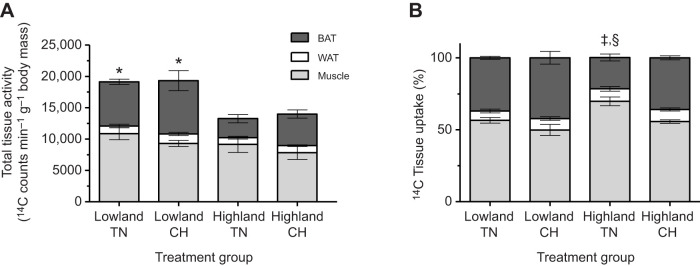
**Uptake of ^14^C-bromopalmitic acid into muscles, white adipose tissue and brown adipose tissue of highland and lowland deer mice exposed to a maximal cold challenge (cold-induced *V̇*_O___2_,max_).** The absolute (A) and relative uptake (B) of ^14^C-bromopalmitic acid into all muscle samples (light grey bar), white adipose tissue (WAT, white bar) and brown adipose tissue (BAT, dark grey bar) of deer mice acclimated to thermoneutral conditions (TN; 30°C, 21 kPa O_2_) or cold hypoxia (CH; 5°C, 12 kPa O_2_). Sample sizes for TN and CH were *N*=6 and *N*=5 for highlanders, and *N*=5 and *N*=6 for lowlanders, respectively. *Main population effect for BAT (*P*<0.05). ^‡^TN highlander muscle and BAT are significantly different than CH highlander muscle and BAT (*post hoc* test: *P*<0.05). ^§^TN highlander muscle and BAT are significantly different from TN lowlander muscle and BAT (*post hoc* test: *P*<0.05). Data are reported as means±s.e.m.

**Fig. 3.**
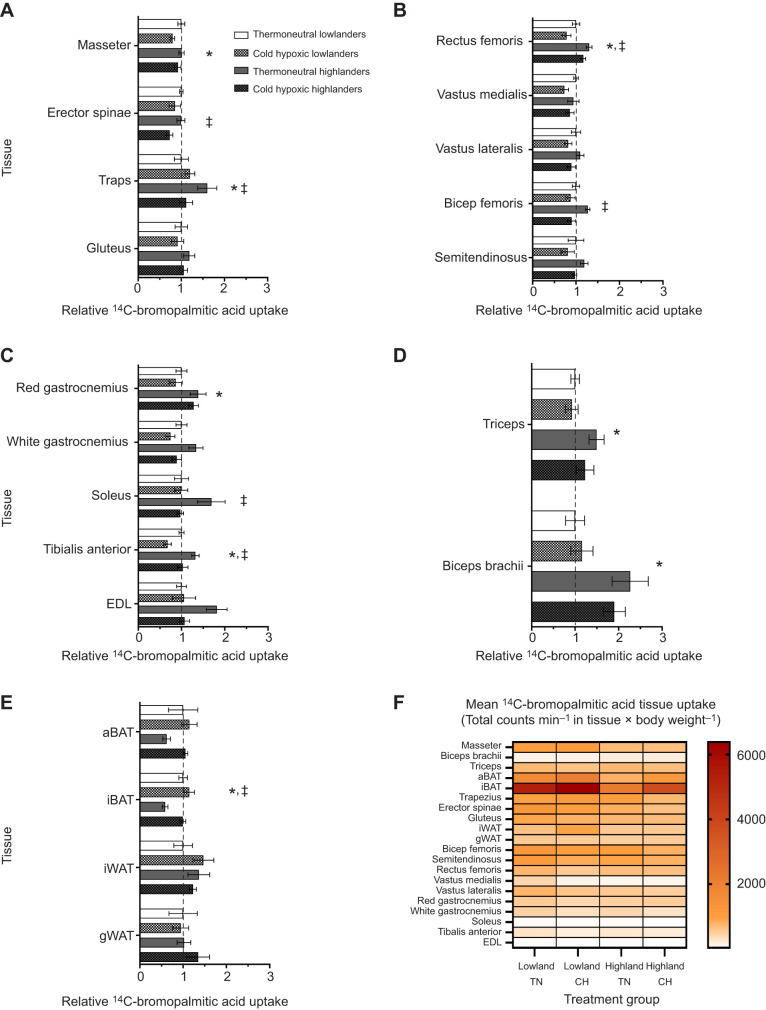
**Uptake of ^14^C-bromopalmitic acid into the individual tissues of deer mice.** Deer mice acclimated to thermoneutral conditions (TN; 30°C, 21 kPa O_2_) or cold hypoxia (CH; 5°C, 12 kPa O_2_) were exposed to a maximal cold challenge in hypoxia (cold-induced *V̇*_O___2_,max_). (A–E) Relative uptake standardized to tissue uptake of TN lowlanders (represented by the dashed line). See Tables S1–S3 for absolute values and sample sizes in central tissues (A); upper hindlimb muscles (B); lower hindlimb muscles (C); forelimb muscles (D); adipose tissues (E). (F) Heat map of the absolute uptake of ^14^C-bromopalmitic acid into individual tissues. *Significant main population effect (*P*<0.05). ^‡^Significant main acclimation effect (*P*<0.05). Data are reported as means±s.e.m. aBAT, auxiliary brown adipose tissue; EDL, extensor digitorum longus; iBAT, interscapular brown adipose tissue; iWAT, interscapular white adipose tissue; gWAT, gonadal white adipose tissue.

We used a radiolabelled tracer fatty acid (^14^C-2-bromopalmitic acid) to determine fatty acid tissue uptake during a maximal cold challenge in deer mice. ^14^C-2-bromopalmitic acid uptake was expressed for each tissue as absolute uptake, as a percentage of total uptake by all sampled tissues (omitting liver, diaphragm, and left and right ventricles due to their much higher uptake) and relative to TN lowland deer mice (Figs 2 and 3, Tables S1–S3).

Fatty acid uptake into all the sampled tissues were categorized into skeletal muscle, BAT and WAT, and were presented as the absolute (Fig. 2A) and uptake as a percentage of total uptake (Fig. 2B) of ^14^C-2-bromopalmitic acid. While there were no significant population (*F*­_1,18_=2.53; *P*=0.13) or acclimation (*F*­_1,18_=2.08; *P*=0.17) effects for the absolute uptake of fatty acids into skeletal muscle (Fig. 2A), there were both population (*F­*_1,18_=11.09; *P*<0.01) and acclimation (*F*­_1,18_=13.10; *P*<0.01) effects for the percentage uptake of fatty acids into skeletal muscle (Fig. 2B). *Post hoc* tests reveal that the proportion of total fatty acid uptake attributed to skeletal muscle was ∼75% in TN-acclimated highlanders, which was significantly greater than in TN-acclimated lowlanders (∼57%; *P*<0.05; Fig. 2B). This population difference is likely to be driven by higher uptake in the following TN-acclimated highlander skeletal muscles: biceps brachii, rectus femoris, red gastrocnemius, tibialis anterior and the triceps (*P*<0.05; Fig. 3, Tables S1,S2). Interestingly, only highlanders showed a decrease in the percentage uptake of radiolabelled fatty acid into skeletal muscles following CH acclimation, with a decline from 75% to 56% of total measured uptake (*P*<0.05). This acclimation response in highlanders was associated with decreases in the percentage uptake of fatty acids in the biceps femoris, erector spinae, masseter, rectus femoris, tibialis anterior and the white gastrocnemius following CH acclimation (*P*<0.05; Fig. 3, Tables S1,S2).

The findings for BAT mirror the results for muscles. There was a significant population effect for the absolute uptake of fatty acids into BAT (Fig. 2A; *F­*_1,18_=13.61; *P*<0.01). There were both significant main population (*F*_1,18_=13.67; *P*<0.01) and acclimation (*F*­_1,18_=11.40; *P*<0.01) effects for the percentage uptake of fatty acids into BAT (Fig. 2B), where fatty acid uptake in BAT (∼16%) was lower in TN-acclimated highlanders compared with TN lowlanders (∼37%; *P*<0.05). However, only in highlanders did BAT show increased fatty acid uptake (by ∼36%) following CH acclimation, which was predominantly driven by increases in uptake by the interscapular BAT depot (*P*<0.05; Fig. 3, Table S3).

Total WAT only contributed ∼8% of total radiolabelled fatty acid uptake, but did not differ between populations (*F*_1,18_=0.87; *P*=0.36) or change with CH acclimation (*F*­_1,18_=0.19; *P*=0.67; Figs 2,3, Table S3).

In general, total absolute ^14^C-fatty acid uptake was greatest in the central organs (liver, heart and diaphragm) and BAT, followed by large trunk muscles located near the centre of the animal (i.e. erector spinae, rectus femoris etc.) compared with the smaller muscles located in the periphery (i.e. gastrocnemius, soleus etc.) as summarized in Tables S1–S4. Both BAT depots and the erector spinae of lowlanders had greater fatty acid total tissue fatty acid uptake compared with highlanders (*P*<0.05; Fig. 3F, Tables S1,S3). Furthermore, total ^14^C-fatty acid uptake decreased with CH acclimation in erector spinae, tibialis anterior and the white gastrocnemius in both populations of deer mice (*P*<0.05; Fig. 3F, Tables S1,S2).

We also determined tissue specific ^14^C activity (counts min^−1^ mg^−1^ tissue), total tissue ^14^C-fatty acid uptake (counts min^−1^×tissue mass g^−1^ body weight) and relative ^14^C fatty acid uptake (% total). In summary, we found that specific ^14^C activity significantly increased following CH acclimation for both auxiliary and interscapular BAT depots and gonadal WAT (*P*<0.05; Table S3), whereas those of the right and left ventricles, liver and diaphragm decreased in both deer mouse populations (*P*<0.05; Table S4). For the individual skeletal muscles, we found that in the soleus, specific activity was greater in lowlanders compared with highlanders (*P*<0.05). White gastrocnemius specific activity in lowlanders was also greater than in highlanders, but only in TN acclimation condition (*P*<0.05). In CH conditions, white gastrocnemius specific activity was greater compared with that in TN conditions, but only for highland deer mice (*P*<0.05). Specific activity of the tibialis anterior showed a unique interaction, where in TN conditions lowlanders had greater activity than highlanders, but the opposite was observed in CH conditions (*P*<0.05). Thus, in the tibialis anterior, specific activity increased with CH acclimation compared with TN for highlanders but decreased for lowlanders (*P*<0.05). Specific activity of the masseter muscle was observed to decrease with CH acclimation, but only in lowland deer mice (*P*<0.05). No other skeletal muscles or tissues demonstrated an effect of population or acclimation for tissue specific activity (*P*>0.05; Tables S1 and S2).

### Tissue enzyme activity

**Fig. 4.**
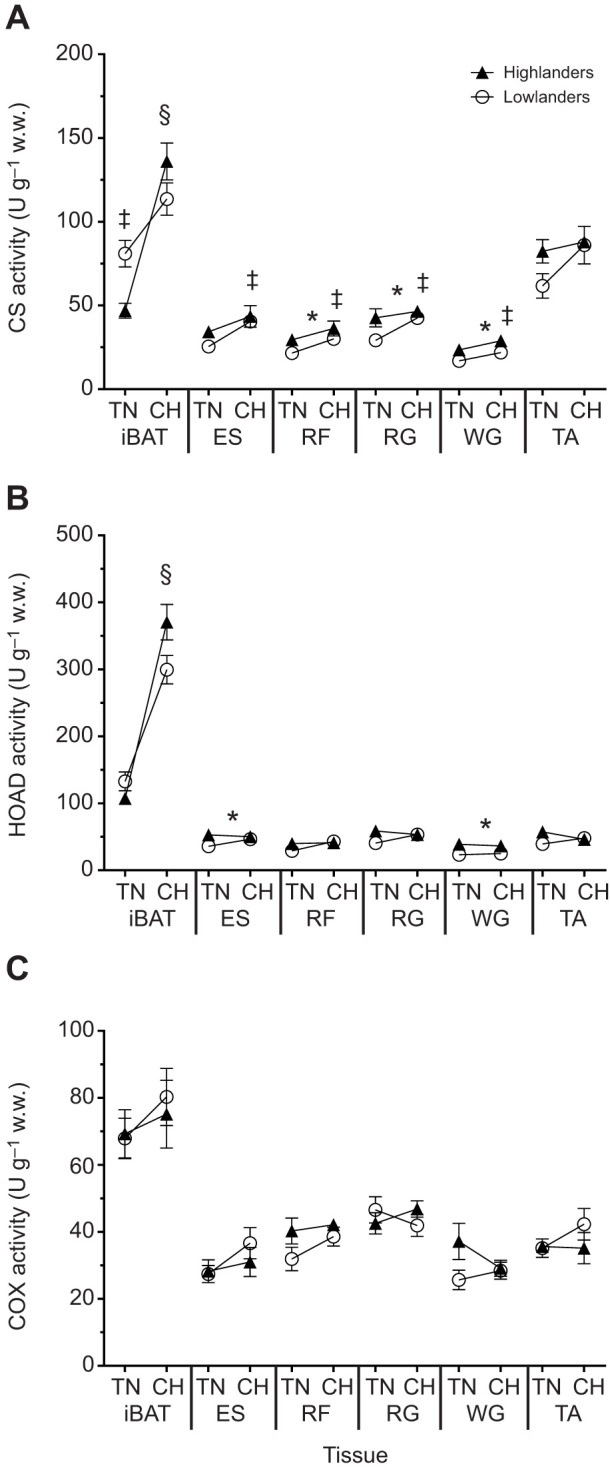
**Apparent maximal enzyme activities (*V*_max_) in selected muscles of deer mice.** Activity of citrate synthase (CS) (A), β-hydroxyacyl-CoA dehydrogenase (HOAD) (B) and cytochrome *c* oxidase (COX) (C) in interscapular brown adipose tissue (iBAT), erector spinae (ES), rectus femoris (RF), red gastrocnemius (RG), white gastrocnemius (WG) and tibialis anterior (TA) of highland and lowland deer mice acclimated to thermoneutral (TN; 30°C, 21 kPa O_2_) or cold hypoxia (CH; 5°C, 12 kPa O_2_) conditions. All enzyme activities were standardized g^−1^ of wet weight (w.w.). Symbols represent significant differences resulting from Holm–Šídák *post hoc* tests (*P*<0.05). *Significant difference between populations. ^‡^Significant difference between acclimations. ^¶^TN highlanders are different from TN lowlanders. ^§^CH highlanders are different from TN highlanders. Sample sizes for TN and CH were *N*=9 and *N*=6 for highlanders, and *N*=9 and *N*=6 for lowlanders, respectively. Data are reported as means±s.e.m.

To assess tissue capacities for aerobic metabolism and fatty acid oxidation, we determined the apparent *V*_max_ for key marker enzymes CS, HOAD and COX in six tissues of interest (iBAT and 5 skeletal muscles; Fig. 4). These tissues were assessed because they demonstrated population differences, and/or showed plasticity with CH acclimation, for fatty acid uptake based on fatty acid tracer results.

The apparent *V*_max_ for CS (Fig. 4A), a marker for mitochondrial volume, was greater in three of the five muscles in highlanders and increased in CH-acclimated deer mice, compared with lowlanders and TN-acclimated mice, respectively. The rectus femoris (population: *F*_1,25_=4.44, *P*=0.04; acclimation: *F*_1,25_=5.10, *P*=0.04), red gastrocnemius (population: *F*_1,25_=4.34, *P*=0.0475; acclimation: *F*_1,25_=4.26, *P*=0.0495), white gastrocnemius (population: *F*_1,26_=11.27, *P*<0.01; acclimation: *F*_1,26_=6.66, *P*=0.02) all showed significant effects of both population and acclimation. The erector spinae displayed a significant effect of acclimation for CS activity, which was higher following CH acclimation in both populations (*F*_1,26_=8.11, *P*<0.01). The tibialis anterior displayed no significant effects of either population (*P*>0.05) or acclimation (*P*>0.05) (Fig. 4A). iBAT displayed a significant population×acclimation interaction (*F*_1,26_=11.95, *P*<0.01), where CS activity in TN-acclimated lowlanders was 73% greater than in TN-acclimated highlanders (*P*<0.05). Additionally, only highlanders showed an increase in iBAT CS activity (up three-fold) upon CH acclimation (*P*<0.05).

The activity of HOAD was measured as a marker for β-oxidation capacity (Fig. 4B; Wickler, 1981). In highlanders, HOAD was ∼23% higher in the erector spinae (*F*_1,26_=4.29, *P*=0.05) and ∼57% higher in the white gastrocnemii (*F*_1,26_=15.10, *P*<0.01) compared with lowland deer mice. No other muscles displayed any significant population effects (*P*>0.05). Likewise, there were no main effects of population on HOAD activity in iBAT (*F*_1,26_=1.87, *P*=0.18). However, there was a significant effect of CH acclimation (*F*_1,26_=167.9, *P*<0.0001). The activity of HOAD in iBAT showed a significant population×acclimation interaction (*F*_1,26_=8.47, *P*<0.01), where CH increased HOAD activity 3.5-fold in highlanders (*P*<0.05). All other tissues measured showed no effect of CH acclimation on HOAD activity (*P*>0.05).

Maximal COX activity was used to assess mitochondrial oxidative capacity (Fig. 4C; Wickler, 1981). There were no significant differences in cytochrome *c* oxidase activities between populations (*P*>0.05) or acclimations (*P*>0.05) among the measured tissues.

### Fatty acid translocase CD36

**Fig. 5.**
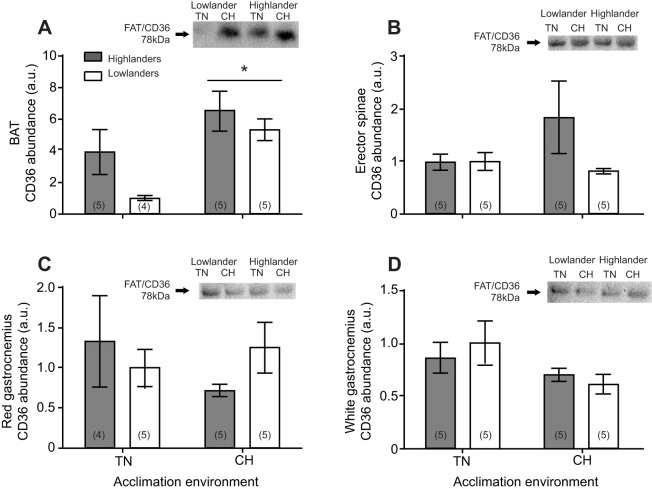
**Relative protein abundance of fatty acid translocase (FAT/CD36) in selected tissues of deer mice.** FAT**/**CD36 levels in (A) interscapular brown adipose tissue (BAT), (B) erector spinae, (C) red gastrocnemius and (D) white gastrocnemius of highland and lowland deer mice acclimated to thermoneutral (TN; 30°C, 21 kPa O_2_) or cold hypoxia (CH; 5°C, 12 kPa O_2_) conditions. Representative western blots are shown for each tissue. *Significant difference between acclimations (*P*<0.05). Sample sizes for each treatment group are shown in parentheses at the bottom of the bar. Data are reported as means±s.e.m.

The capacity for fatty acid uptake in iBAT, erector spinae, and both red and white gastrocnemii was evaluated by quantifying the total protein abundance of FAT/CD36 in whole tissue homogenates. CH acclimation led to a significant increase in FAT/CD36 protein abundance in iBAT (*F*_1,15_=12.14, *P*<0.05). Abundance of FAT/CD36 trended to be higher in the iBAT of highlanders compared with lowlanders but failed to reach the level of statistical significance (effects of population, *F*_1,15_=4.25, *P*=0.06) (Fig. 5A). For the erector spinae, we found no significant effects of population (*F*_1,16_=1.93, *P*=0.18) or acclimation (*F*_1,16_=0.84, *P*=0.37) on FAT/CD36 protein abundance (Fig. 5B). Similarly, both red and white gastrocnemii displayed no differences in FAT/CD36 abundance between populations (*F*_1,15_=0.11, *P*=0.75 and *F*_1,16_=0.04, *P*=0.85, respectively) or with acclimation (*F*_1,15_=0.33, *P*=0.57 and *F*_1,16_=3.82, *P*=0.07, respectively) (Fig. 5C,D).

## DISCUSSION

The goal of this study was to determine tissue uptake of circulatory fatty acids during maximal rates of thermogenesis in deer mice from low and high altitude. We hypothesized that the tissue distribution of fatty acid uptake would be affected by CH acclimation in highlanders with increased uptake by BAT. We found that circulatory fatty acid uptake at cold-induced *V̇*_O___2_,max_ could be separated into tissues with high (liver, heart, diaphragm, iBAT), moderate (auxiliary BAT, erector spinae, masseter, thigh and hamstring muscles) and low (WAT depots and small skeletal muscles located on the extremities of the forelimb and hindlimb) uptake. When fatty acid uptake from all the skeletal muscles was combined and compared with that of BAT, we found that CH acclimation led to a shift towards greater BAT uptake, but only in highland deer mice, supporting our hypothesis. This was consistent with increases in whole animal lipid oxidation (Fig. 1C) and previous data showing NST increases with CH acclimation in highlanders, but not lowlanders (Coulson et al., 2021). Greater fatty acid uptake into BAT with CH in highlanders was associated with increases in total BAT FAT/CD36 protein abundance. Interestingly, BAT from highlanders also had higher activities of CS and HOAD compared with lowlanders, which further increased with CH acclimation. This suggests a greater sink for lipid oxidation resulting in increased capacity for BAT fatty acid uptake from the circulation. In three of the five skeletal muscles where we quantified enzyme activities, highlanders showed higher CS activity compared with lowlanders. After CH acclimation, there was an increase in CS in these same muscles, suggesting environmentally induced plasticity with greater mitochondrial densities compared with TN acclimation. Two muscles, the erector spinae and white gastrocnemius, also showed increases in HOAD activities with CH, but only in highlanders. Interestingly, these differences in muscle phenotype were not directly associated with variation in muscle fatty acid uptake. These results show that tissue uptake of circulatory fatty acids varies during peak thermogenesis, both between lowland and highland populations of deer mice, but also with CH acclimation simulating high altitude conditions at 4300 m. Using a comprehensive sampling of tissues, we were able to demonstrate a coordinated redistribution of fatty acid uptake from skeletal muscles to BAT in highland deer mice exposed to conditions experienced in their natural alpine environment.

We found that thermogenic capacity in hypoxia was greater in highland deer mice compared with lowland deer mice, consistent with findings of our previous work (Lyons et al., 2021). Highlanders also showed a significant increase in cold-induced *V̇*_O___2_,max_ in response to CH acclimation, whereas lowland deer mice did not (Fig. 1A). We used a TN acclimation (30°C, 21 kPa O_2_) to ensure mice were housed within their thermoneutral zone (Hayward et al., 2022). When both populations were acclimated to TN conditions, *post hoc* testing revealed no significant differences in cold-induced *V̇*_O___2_,max_. However, *V̇*_O___2_,max_ values were lower than we previously reported for either population housed in standard normoxia (Lyons et al., 2021), demonstrating that common mouse holding conditions of 23°C represent a mild cold acclimation (McKie et al., 2019). Similarly, we found that lipid oxidation rates were lower in TN-acclimated deer mice than previously reported for deer mice kept in standard normoxia holding conditions (Lyons et al., 2021). Highland deer mice showed a significant increase in whole-animal lipid oxidation rates with CH acclimation (Fig. 1C), associated with the increase in *V̇*_O___2_,max_; a response not shared by lowland deer mice. This increase in lipid oxidation is consistent with the response observed when highlanders are acclimated to cold alone (Hayward et al., 2022). In lowland deer mice, the addition of hypoxia may antagonize any cold-induced increases in whole-animal lipid oxidation, similarly to the response observed for NST in lab mice (Beaudry and McClelland, 2010). However, highlanders have evolved a distinct plasticity response to combined cold hypoxia.

High rates of lipid oxidation in highland deer mice during hypoxic cold-induced *V̇*_O___2_,max_ are possible due to the elevated circulatory delivery of fatty acids (Lyons and McClelland, 2022), but their tissue destination was unclear. Previous work on anesthetized rats examined fatty acid uptake in a few tissues following acute cooling. However, this probably does not reflect tissue uptake of fatty acids during maximal rates of heat production in conscious, freely behaving mice. Radiolabelled 2-bromopalmitic acid has previously been used to trace uptake of fatty acids into tissues in rodents (Oakes et al., 1999; 2006; Furler et al., 2000; Hegarty et al., 2002; Hauton et al., 2009; Schönke et al., 2018). This technique also allowed us to determine fatty acid uptake across multiple tissues and is one of the first comprehensive analyses of tissue activity during high rates of thermogenesis in mice. It is important to note that while using this non-oxidizable tracer fatty acid provides information for fatty acid tissue localization, it does not indicate its fate once trapped inside the cell (i.e. intracellular storage or mitochondrial oxidation). Despite this limitation, there is clear evidence to suggest that with cold acclimation and cold exposure, increased fatty acid uptake directly correlates with increased fatty acid oxidation, especially in thermo-active tissue, such as BAT (Hauton et al., 2009; U Din et al., 2016). While it is possible that ^14^C-bromopalmitate taken up by tissues may have been destined for intracellular storage, this seems unlikely for the deer mice in this study, as they were exposed to maximal cold challenges. Thus, the fate of fatty acids would predominantly be used in tissue mitochondrial oxidation to power increased heat production.

We found that a large proportion of total fatty acid uptake occurred in central tissues (liver, heart and diaphragm) and in BAT. In addition, individual muscles that are larger and closer to the core of the animal (i.e. erector spinae, rectus femoris, bicep femoris) take up more fatty acids than smaller muscles located near the extremities (i.e. biceps brachii, soleus, tibialis anterior; Fig. 1). Our findings suggest that with increased demand for heat production, blood flow is shunted away from the peripheral muscles and towards tissues located near the core of the animal. For example, the trunk muscles are generally larger compared with the muscles found at the extremities. The heat output from these larger trunk muscles most likely aids with maintaining stable temperatures of the surrounding vital organs. Therefore, during thermogenesis, these large central tissues take up fatty acids to a greater extent compared with those found in the extremity. We also observed a redistribution of tissue fatty acid uptake with CH acclimation. CH-acclimated highland deer mice showed a decline in muscle uptake and an increase in BAT uptake of fatty acids compared with TN highlanders (Figs 2,3). In contrast, lowland deer mice showed no change in the relative fatty acid uptake between muscle and BAT with CH. Previous findings demonstrate that in rats, BAT increases both fatty acid uptake and oxidation following acclimation to cold alone, with no changes in uptake at other organs or muscle tissue (Hauton et al., 2009). This shift in fatty acid uptake in highlanders, from muscle to BAT, may be associated with a redirection of blood flow with CH, as observed in cold-acclimated rats when exposed to an acute cold challenge (Foster and Frydman, 1979). The fact that lowland deer mice did not show a redistribution of fatty acid uptake in CH may reflect an antagonistic influence of hypoxia on the effects of chronic cold observed in other lowland native rodents (Beaudry and McClelland, 2010).

BAT is crucial for thermogenesis in small eutherian mammals as it is the main site of NST and can contribute to over 50% of total thermogenic capacity (Foster and Frydman, 1979; Van Sant and Hammond, 2008). Upon cold exposure, fatty acids activate mitochondrial UCP-1, the mitochondrial membrane protein facilitating NST, which increases BAT metabolism (Cannon and Nedergaard, 2004). Triglyceride-rich lipoproteins and non-esterified fatty acids provide the main substrates for sustaining the increased BAT activity (Bartelt et al., 2011). Only in highlanders was there an increase in circulating fatty acid uptake into BAT depots with acclimation, from 16% in TN to 36% in CH (Fig. 2B), driven by the uptake of fatty acids into the iBAT rather than the auxiliary BAT (aBAT) depot (Fig. 3E,F). These findings are consistent with increased NST in highland, but not lowland, deer mice after CH acclimation, associated with increases in UCP-1 expression, and iBAT mitochondrial respiration capacity (Coulson et al., 2021). While we found the capacity for fatty acid transport into iBAT via FAT/CD36 increased after CH acclimation, there were no differences between the populations (Fig. 5A), consistent with our previous findings (Lyons and McClelland, 2022). The increase in fatty acid uptake in the BAT of CH highlanders does not directly correlate to increases in BAT FAT/CD36, suggesting that BAT fatty acid uptake may be limited by blood flow and fatty acid delivery in lowlanders, as we have proposed previously (Lyons and McClelland, 2022). However, CH-acclimated highlanders had elevated CS and HOAD activities in iBAT (Fig. 4A,B), suggesting an increased mitochondrial density and β-oxidation capacity compared with iBAT in lowland deer mice. Altogether, these findings for BAT help explain the unique CH-acclimated increase in whole-animal lipid oxidation rates observed in thermoregulating highland deer mice (Fig. 1C; Lyons et al., 2021).

Muscle recruitment for shivering thermogenesis are known to increase rates of fatty acid oxidation (Blondin et al., 2014). Interestingly, rats acclimated to warm conditions exposed to an acute cold challenge have minimal BAT activity and rely predominately on shivering for heat production (Vaillancourt et al., 2009). In this study, we used a TN acclimation to minimize BAT activity, which was generally associated with reduced CS and HOAD activities in iBAT compared with CH (Fig. 4). We found that TN-acclimated highlanders had a higher relative uptake of ^14^C-bromopalmitic acid into skeletal muscles (∼75% of total uptake) compared to TN-acclimated lowlanders (∼57%, Fig. 2), despite similar whole-animal lipid oxidation rates during hypoxic cold-induced *V̇*_O___2_,max_. While differences in fatty acid uptake may be associated with intramuscular stores of triglycerides (IMTGs), our previous findings show no population differences in gastrocnemius IMTG, at rest or following cold-induced VO_2_max (Lyons and McClelland, 2022). However, whether the same is true for other tissues is currently unclear. Therefore, our current findings suggest that rates of circulating fat use by shivering muscle is greater for highland deer mice, at least with TN acclimation, compared with lowlanders. A higher cardiac output in highlanders (Tate et al., 2020), in combination with an higher capillarity of highland muscle (Lui et al., 2015; McClelland and Scott, 2019) and increased rate of circulatory fatty acid delivery (Lyons and McClelland, 2022) may account for these observations. Although capacity for muscle fatty acid uptake via CD36/FAT did not differ between populations, the elevated oxidative phenotype of highlander muscle would allow for increased capacities for lipid oxidation (Fig. 4, Cheviron et al., 2012; Lui et al., 2015; Lau et al., 2017; Lyons and McClelland, 2022).

There are two major limitations associated with thermogenesis at altitude: (1) the inhibitory effect of chronic hypoxia on BAT activity (Beaudry and McClelland, 2010; Coulson et al., 2021) and (2) the limitation of O_2_ and substrate transport to thermo-effector tissues in hypoxia (reviewed in McClelland and Scott, 2019). Highland deer mice have evolved mechanisms to overcome both of these limitations. Whole-animal lipid oxidation rates during cold-induced *V̇*_O___2_,max_ in hypoxia were not changed by CH acclimation in lowland deer mice (Fig. 1C), consistent with previous work demonstrating no changes in NST after CH acclimation (Coulson et al., 2021). Unlike results seen in the highlanders, in lowlanders there were no shifts in fatty acid uptake from muscle to BAT following CH acclimation (Fig. 2) and no changes in iBAT CS activity with CH (Fig. 4). These findings suggest that lowland deer mice respond to CH similarly to lab mice (Beaudry and McClelland, 2010), where hypoxia has an antagonistic effect on cold-induced changes in thermogenesis and fatty acid uptake. Furthermore, acclimation to CH conditions may be hindering lowlanders' ability to transport O_2_ and metabolic substrate to thermo-effector tissue during heat production, limiting the rate lipids can be metabolised (Beaudry and McClelland, 2010; McClelland and Scott, 2019). However, highlanders show a cold acclimation response regardless of environmental oxygen availability, likely attributed to their evolved capacities for greater O_2_ and substrate transport (McClelland and Scott, 2019). Therefore, the blunted BAT activity and the impeded O_2_ delivery associated with hypoxia during acclimation to simulated high-altitude conditions may explain why lowlanders have lower thermogenic lipid oxidation rates compared with highlanders. More work is needed to identify how cold and hypoxia interact to impact BAT activity in deer mice.

### Conclusions

Highland deer mice have evolved impressive thermoregulatory capacities in hypoxia to ensure survival in the challenging environment of high altitude. Altogether, our findings suggest that highland deer mice acclimated to simulated high-altitude conditions increase maximal heat production with a corresponding increase in lipid metabolism, when compared with lowlanders, consistent with previous studies (Tate et al., 2020; Lyons et al., 2021). These findings are associated with the increased cardiac output (Tate et al., 2017, 2020), circulatory fat delivery rates (Lyons and McClelland, 2022), capillarity and oxidative phenotype of muscle (Lui et al., 2015; Lau et al., 2017; Mahalingam et al., 2020; Lyons and McClelland, 2022), and aerobic capacity of iBAT (Coulson et al., 2021; Hayward et al., 2022) of highland deer mice. While there has been emerging evidence for the increased role of circulatory lipids for powering thermogenesis in thermo-effector tissue (Lyons and McClelland, 2022), the specific distribution of these circulatory lipids had yet to be determined in detail.

In this study, we determined fatty acid uptake among 24 tissues during peak thermogenesis in both highland and lowland deer mice acclimated to either TN or CH conditions. Our study emphasizes that in deer mice, larger and more centralized tissues, such as the liver, iBAT and rectus femoris, uptake more circulatory fatty acids than smaller muscles located near the extremity of the animal, such as the soleus and biceps brachii. We show that at cold-induced *V̇*_O___2_,max_, CH acclimation increases fatty acid uptake from the circulation into BAT to a greater extent in highlanders. Our results provide supporting evidence for BAT's essential role in enhancing heat production by increasing NST, while the importance of shivering thermogenesis is decreased (Blondin et al., 2014; Jastroch et al., 2021). However, while individual muscles may not be as active as BAT for fatty acid uptake, collectively, total muscle may provide a larger source of circulatory fatty acid clearance during heat production compared with BAT (>50%, Fig. 2; Blondin et al., 2017; Saari et al., 2020). These high clearance rates of fatty acids are presumably to sustain shivering and contribute to the high rates of lipid oxidation observed in deer mice (Lyons et al., 2021; Lyons and McClelland, 2022). Lastly, we propose that the high rates of lipid oxidation observed in thermoregulating highland deer mice are associated with adaptations allowing them to overcome the hindering effects hypoxia has on O_2_ transport (McClelland and Scott, 2019) and BAT activity (Beaudry and McClelland, 2010; Coulson et al., 2021). Highland deer mice have adapted to deliver and take up circulatory lipids more effectively compared with their lowland conspecifics. This increased lipid uptake capacity is primarily associated with the greater tissue capillarity and higher cardiac output in highlanders (Tate et al., 2020), leading to higher rates of lipid delivery to thermo-effector tissues (Lyons and McClelland, 2022). Future research should continue to distinguish the contribution of circulating lipids and intracellular lipids in both BAT and muscles in the context of total heat production in highland native deer mice.

## Supplementary Material

10.1242/jexbio.247340_sup1 Supplementary information
