# Supplementary material for: Highland deer mice support increased thermogenesis in response to chronic cold hypoxia by shifting uptake of circulating fatty acids from muscles to brown adipose tissue
Source: J Exp Biol. 2024 Apr 11;227(7):jeb247340. doi: 10.1242/jeb.247340 (PMC11057874; doi:10.1242/jeb.247340)
Supplement: Supplementary information [file jexbio-227-247340-s1.pdf]

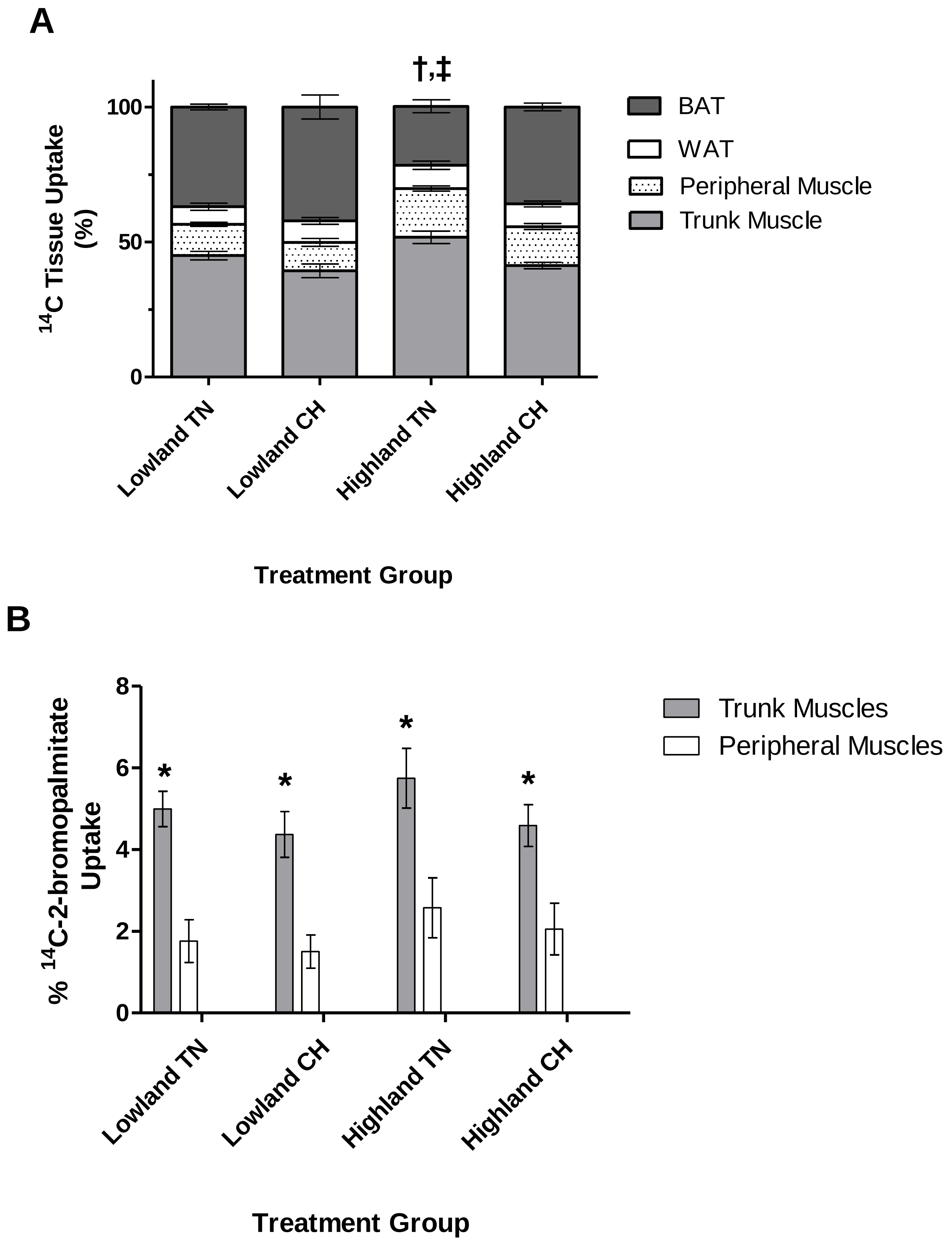
A
†,‡
100
50
0
^{14}C Tissue Uptake (%)
BAT
WAT
Peripheral Muscle
Trunk Muscle
Lowland TN
Lowland CH
Highland TN
Highland CH
Treatment Group
B
8
6
4
2
0
% ^{14}C-2-bromopalmitate Uptake
*
Trunk Muscles
Peripheral Muscles
Treatment Group

Fig. S1. A- The combined relative uptake of ^{14}C-bromopalmitic acid into all trunk muscles (light grey bar), all peripheral muscles (dotted white bar), white adipose tissue (WAT, white bar) and brown adipose tissue (BAT, dark grey bar). B-The average relative uptake of ^{14}C-bromopalmitic acid into an individual trunk muscle (grey bar) is greater than uptake into an individual peripheral muscle (white bar). Highland and lowland deer mice were acclimated to thermoneutral conditions (TN, 30ºC, 21kPa O_2) or cold hypoxia (CH, 5ºC, 12kPa O_2) and exposed to a maximal cold challenge in hypoxia. "Trunk" muscles consist of the following: masseter, erector spinae, trapezius, gluteus, rectus femoris, vastus lateralis, vastus medialis, bicep femoris, and semitendinosus. "Peripheral" muscles consist of the following: red gastrocnemius, white gastrocnemius, soleus, tibialis anterior, extensor digitorum longus, bicep brachii, and triceps. ‡TN highlander trunk and peripheral muscle and BAT are significantly different than CH highlander trunk and peripheral muscle and BAT (post-test: $P < 0.05$). † TN highlander trunk and peripheral muscle and BAT are significantly different than TN lowlander trunk and peripheral muscle and BAT (post-test: $P < 0.05$). *Significantly different from peripheral muscles ($P < 0.05$). Data are presented as means ± s.e.m.

Table S1. Central core and upper hindlimb muscle average mass (mg), specific tissue activity (^{14}C-counts per minute (CPM) per mg tissue), total tissue activity (total tissue CPM per g body mass), and relative tissue uptake of ^{14}C-palmitic acid (as % of total tissue CPM) of highland and lowland deer mice acclimated to thermoneutral conditions (TN; 30ºC, 21kPa O_2) or cold hypoxia (CH; 5ºC, 12kPa O_2) exposed to a maximal cold challenge in hypoxia (cold-induced $\dot{V}O_2$max).

**Tissue**	**Tissue mass (mg)**				**Specific activity (CPM mg $^{-1}$)**				**Total activity (CPM g $^{-1}$ body weight)**				**Relative tissue uptake (%)**			
	Highlanders		Lowlanders		Highlanders		Lowlanders		Highlanders		Lowlanders		Highlanders		Lowlanders	
Central/Core Muscles	TN	CH	TN	CH	TN	CH	TN	CH	TN	CH	TN	CH	TN	CH	TN	CH
Masseter	75.6 ± 2.7 (6)	80.0 ± 6.2 (5)	66.0 ± 4.1 (5)	**90.9 ± 5.6‡ (6)**	8.2 ± 1.0 (6)	8.2 ± 1.3 (5)	12.8 ± 2.0 (5)	**6.8 ± 1.2‡ (5)**	717.6 ± 130.2 (6)	676.9 ± 94.0 (5)	1027.0 ± 153.1 (5)	759.2 ± 91.6 (5)	5.28 ± 0.32 (6)	**4.84 ± 0.38# (5)**	5.26 ± 0.42 (5)	**4.18 ± 0.28# (5)**
Erector Spinae	85.3 ± 5.0 (6)	72.3 ± 10.4 (5)	89.9 ± 2.5 (5)	98.0 ± 9.8 (6)	7.8 ± 0.6 (6)	11.4 ± 2.7 (5)	9.2 ± 1.1 (5)	7.8 ± 1.0 (6)	**848.0 ± 93.9* (6)**	**574.1 ± 30.8*# (4)**	1288.9 ± 127.7 (5)	**1042.7 ± 133.9# (6)**	6.63 ± 0.61 (6)	**4.88 ± 0.50# (5)**	6.68 ± 0.28 (5)	**5.69 ± 0.89# (6)**
Trapezius	69.6 ± 6.1 (6)	49.8 ± 8.3 (5)	55.6 ± 4.8 (5)	68.0 ± 4.1 (6)	14.4 ± 1.8 (6)	31.3 ± 9.5 (5)	18.5 ± 3.7 (5)	17.2 ± 1.7 (6)	820.6 ± 105.2 (5)	755.1 ± 106.6 (5)	983.0 ± 221.1 (5)	1173.1 ± 186.9 (6)	8.00 ± 1.10 (6)	5.56 ± 0.76 (5)	5.00 ± 0.82 (5)	6.00 ± 0.56 (6)
Gluteus	94.1 ± 5.0 (6)	99.9 ± 14.8 (5)	68.8 ± 3.2 (4)	98.3 ± 10.9 (6)	5.8 ± 0.8 (6)	6.5 ± 1.5 (5)	9.3 ± 1.4 (5)	6.2 ± 0.8 (6)	758.0 ± 115.9 (6)	708.1 ± 79.8 (5)	941.1 ± 136.7 (5)	811.5 ± 80.4 (6)	5.82 ± 0.63 (6)	5.19 ± 0.44 (5)	4.91 ± 0.71 (5)	4.48 ± 0.70 (6)
Upper Hindlimb Muscles																
Rectus Femoris	**77.0 ± 3.9* (6)**	**69.0 ± 7.1* (5)**	58.9 ± 6.6 (5)	64.0 ± 4.5 (6)	8.2 ± 1.5 (6)	12.0 ± 3.0 (5)	14.7 ± 3.8 (5)	10.3 ± 1.7 (6)	695.6 ± 113.0 (6)	666.9 ± 104.5 (5)	768.2 ± 79.5 (5)	578.8 ± 67.2 (6)	**5.22 ± 0.27* (6)**	**4.68 ± 0.22*# (5)**	4.00 ± 0.34 (5)	**3.14 ± 0.40# (6)**
Vastus Lateralis	62.1 ± 4.0 (6)	54.7 ± 6.4 (5)	59.8 ± 6.3 (5)	61.6 ± 2.6 (6)	9.8 ± 0.4 (6)	15.1 ± 4.8 (5)	10.4 ± 0.5 (4)	11.4 ± 1.4 (6)	575.1 ± 82.6 (6)	517.8 ± 95.0 (5)	778.5 ± 104.8 (5)	551.8 ± 20.5 (5)	4.41 ± 0.36 (6)	3.60 ± 0.40 (5)	4.03 ± 0.43 (5)	3.31 ± 0.34 (6)
Vastus Medialis	13.1 ± 1.6 (6)	11.0 ± 1.4 (5)	13.2 ± 1.2 (5)	11.0 ± 0.9 (6)	54.5 ± 5.6 (6)	92.8 ± 21.7 (5)	80.4 ± 20.7 (4)	88.7 ± 10.2 (6)	136.9 ± 29.1 (6)	137.1 ± 31.4 (5)	217.7 ± 18.2 (4)	156.5 ± 30.4 (6)	1.02 ± 0.14 (6)	0.93 ± 0.11 (5)	1.09 ± 0.06 (4)	0.80 ± 0.11 (6)
Bicep Femoris	**142.9 ± 9.3† (6)**	**106.5 ± 12.3‡ (5)**	106.1 ± 5.7 (5)	117.3 ± 7.7 (6)	3.8 ± 0.6 (6)	5.8 ± 1.3 (5)	6.5 ± 1.1 (5)	5.3 ± 0.5 (6)	1085.7 ± 168.9 (6)	819.8 ± 155.5 (5)	1250.1 ± 173.8 (5)	1044.3 ± 123.7 (6)	8.15 ± 0.31 (6)	**5.76 ± 0.62# (5)**	6.42 ± 0.53 (5)	**5.60 ± 0.69# (6)**
Semitendinosus	128.2 ± 4.9 (6)	106.5 ± 10.5 (5)	95.3 ± 15.4 (5)	105.2 ± 10.4 (6)	3.9 ± 0.63 (6)	6.2 ± 1.6 (5)	6.2 ± 0.6 (4)	5.6 ± 0.5 (6)	954.8 ± 146.1 (6)	826.0 ± 117.0 (5)	1135.5 ± 180.8 (5)	869.5 ± 111.1 (6)	7.20 ± 0.53 (6)	5.88 ± 0.26 (5)	6.05 ± 1.09 (5)	4.89 ± 0.93 (6)

Data are presented as means ± s.e.m., with sample sizes in parentheses. Bolded values indicate statistical significance. *Significant main population effect ($P < 0.05$). # Significant main acclimation effect ($P < 0.05$). Significant differences in population × acclimation interactions result from Holm Sidak post hoc tests ($P < 0.05$). †Significantly different than lowlanders, within an acclimation. ‡Significantly different than thermoneutral conditions, within a population. Statistics summary of F and P values for 2-way ANOVA for population, acclimation, and population × acclimation interaction is found in Table S5.

Journal of Experimental Biology • Supplementary information

Table S2. Lower hindlimb and forelimb muscle average mass (mg), specific tissue activity (^{14}C-counts per minute (CPM) per mg tissue), total tissue activity (total tissue CPM per g body mass), and relative tissue uptake of ^{14}C-palmitic acid (as % of total tissue CPM) of highland and lowland deer mice acclimated to thermoneutral conditions (TN; 30ºC, 21kPa O_2) or cold hypoxia (CH; 5ºC, 12kPa O_2) exposed to a maximal cold challenge in hypoxia (cold-induced $\dot{V}O_{2}max$).

**Tissue**	**Tissue mass (mg)**				**Specific activity (CPM mg $^{-1}$)**				**Total activity (CPM g $^{-1}$ body weight)**				**Relative tissue uptake (%)**			
	Highlanders		Lowlanders		Highlanders		Lowlanders		Highlanders		Lowlanders		Highlanders		Lowlanders	
Lower Hindlimb Muscles	TN	CH	TN	CH	TN	CH	TN	CH	TN	CH	TN	CH	TN	CH	TN	CH
Red Gastrocnemius	**37.5 ± 3.4* (6)**	**41.0 ± 2.6* (5)**	31.5 ± 5.2 (5)	28.5 ± 1.3 (5)	26.3 ± 4.1 (6)	24.7 ± 5.4 (5)	41.6 ± 13.9 (5)	32.2 ± 4.0 (6)	511.6 ± 70.8 (6)	542.8 ± 108.6 (5)	562.9 ± 75.3 (5)	447.9 ± 32.4 (6)	**4.05 ± 0.54* (6)**	**3.74 ± 0.33* (5)**	2.93 ± 0.37 (5)	2.53 ± 0.45 (6)
White Gastrocnemius	**56.4 ± 4.0† (6)**	42.2 ± 6.1 (5)	37.7 ± 0.7 (4)	42.2 ± 3.4 (6)	**9.3 ± 1.1† (6)**	**15.6 ± 3.8‡ (5)**	17.3 ± 2.4 (5)	13.9 ± 1.4 (6)	449.5 ± 72.3 (6)	**315.0 ± 57.5# (5)**	498.1 ± 88.2 (5)	**345.6 ± 33.7# (6)**	3.41 ± 0.42 (6)	**2.27 ± 0.28# (5)**	2.55 ± 0.33 (5)	**1.90 ± 0.26# (6)**
Soleus	5.5 ± 0.4 (6)	4.8 ± 0.8 (5)	3.5 ± 0.2 (3)	4.6 ± 0.5 (6)	**159.3 ± 16.2* (6)**	**192.5 ± 48.5* (5)**	307.9 ± 71.1 (3)	220.7 ± 34.9 (6)	71.4 ± 9.4 (6)	46.7 ± 5.5 (5)	70.5 ± 17.3 (3)	62.8 ± 7.3 (6)	0.59 ± 0.11 (6)	0.34 ± 0.03 (5)	0.35 ± 0.06 (3)	0.35 ± 0.05 (6)
Tibialis Anterior	32.1 ± 2.0 (6)	27.4 ± 3.5 (5)	26.0 ± 3.6 (4)	28.7 ± 1.9 (6)	**18.0 ± 2.4† (6)**	**26.2 ± 6.6†‡ (5)**	30.9 ± 8.9 (4)	**16.6 ± 1.3‡ (6)**	273.7 ± 41.2 (6)	**227.5 ± 44.2# (5)**	307.1 ± 29.5 (4)	**195.8 ± 19.8# (6)**	**2.08 ± 0.13* (6)**	**1.62 ± 0.19*# (5)**	1.57 ± 0.09 (4)	**1.06 ± 0.14# (6)**
Extensor Digitorum Longus	5.9 ± 0.5 (6)	4.1 ± 0.5 (5)	4.4 ± 0.9 (5)	4.0 ± 0.3 (5)	120.8 ± 23.8 (6)	178.0 ± 40.0 (5)	206.9 ± 41.5 (5)	149.7 ± 21.0 (6)	59.4 ± 12.2 (6)	37.4 ± 7.3 (5)	59.4 ± 13.3 (5)	48.7 ± 14.0 (6)	0.44 ± 0.06 (6)	0.26 ± 0.03 (5)	0.24 ± 0.03 (4)	0.26 ± 0.07 (6)
Forelimb Muscles																
Bicep Brachii	**17.2 ± 1.9* (6)**	**16.2 ± 1.0* (5)**	10.0 ± 1.7 (3)	13.3 ± 1.4 (6)	54.3 ± 9.6 (6)	56.3 ± 5.7 (5)	90.3 ± 27.3 (3)	61.0 ± 7.3 (6)	220.9 ± 47.8 (6)	210.3 ± 50.1 (5)	150.6 ± 37.9 (3)	155.6 ± 29.6 (6)	**1.72 ± 0.31* (6)**	**1.44 ± 0.19* (5)**	0.76 ± 0.17 (3)	0.88 ± 0.19 (6)
Triceps	68.5 ± 3.1 (6)	66.2 ± 8.0 (5)	53.8 ± 4.5 (5)	63.1 ± 5.2 (6)	10.5 ± 1.1 (6)	12.1 ± 2.4 (5)	14.9 ± 1.5 (5)	11.4 ± 1.1 (6)	737.5 ± 99.3 (6)	684.3 ± 179.2 (5)	742.5 ± 100.7 (5)	644.7 ± 77.7 (6)	**5.76 ± 0.67* (6)**	**4.74 ± 0.79* (5)**	3.86 ± 0.38 (5)	3.57 ± 0.56 (6)

Data are presented as means ± s.e.m., with sample sizes in parentheses. Bolded values indicate statistical significance. *Significant main population effect (P <0.05). # Significant main acclimation effect (P<0.05). Significant differences in population × acclimation interactions result from Holm Sidak post hoc tests (P < 0.05). †Significantly different than lowlanders, within an acclimation. ‡Significantly different than thermoneutral conditions, within a population. Statistics summary of F and P values for 2-way ANOVA for population, acclimation, and population × acclimation interaction is found in Table S5.

Table S3. Brown adipose tissue (BAT) and white adipose tissue (WAT) average mass (mg), specific tissue activity (^{14}C-counts per minute (CPM) per mg tissue), total tissue activity (total tissue CPM per g body mass), and relative tissue uptake of ^{14}C-palmitic acid (as % of total tissue CPM) of highland and lowland deer mice acclimated to thermoneutral conditions (TN; 30°C, 21kPa O_2) or cold hypoxia (CH; 5°C, 12kPa O_2) exposed to a maximal cold challenge in hypoxia (cold-induced $\dot{V}O_2$max).

Tissue	Tissue mass (mg)				Specific activity (CPM mg $^{-1}$)				Total activity (CPM g $^{-1}$ body weight)				Relative tissue uptake (%)			
	Highlanders		Lowlanders		Highlanders		Lowlanders		Highlanders		Lowlanders		Highlanders		Lowlanders	
Brown Adipose Tissue (BAT)	TN	CH	TN	CH	TN	CH	TN	CH	TN	CH	TN	CH	TN	CH	TN	CH
Auxiliary BAT	32.8 ± 3.4 (6)	25.7 ± 4.4 (5)	35.1 ± 6.4 (5)	30.1 ± 5.1 (6)	56.9 ± 16.0 (6)	**109.3 ± 20.3# (4)**	59.1 ± 18.5 (4)	**189.0 ± 44.9# (6)**	**814.2 ± 214.2* (6)**	**1237.9 ± 288.2* (5)**	1701.4 ± 512.4 (5)	2117.8 ± 477.0 (6)	5.73 ± 0.82 (6)	9.76 ± 0.50 (4)	9.26 ± 3.13 (5)	10.61 ± 1.64 (6)
Interscapular BAT	108.3 ± 13.2 (6)	82.5 ± 9.0 (5)	111.1 ± 19.0 (5)	108.3 ± 13.2 (6)	20.0 ± 9.2 (6)	**49.3 ± 14.3# (5)**	21.3 ± 4.7 (4)	**47.9 ± 9.0# (6)**	**2225.0 ± 461.5* (6)**	**3780.2 ± 447.4* (5)**	5338.4 ± 683.9 (5)	6381.4 ± 1191.0 (6)	**16.12 ± 1.82* (6)**	**27.45 ± 1.87*# (5)**	27.66 ± 2.75 (5)	**31.59 ± 3.37# (6)**
White Adipose Tissue (WAT)																
Inguinal WAT	112.1 ± 20.0 (5)	163.3 ± 42.2 (5)	84.9 ± 29.9 (4)	89.0 ± 20.1 (6)	3.2 ± 1.2 (6)	1.8 ± 0.8 (4)	2.4 ± 1.0 (4)	13.5 ± 4.9 (6)	585.2 ± 138.5 (6)	575.6 ± 63.0 (5)	655.1 ± 148.7 (5)	962.0 ± 160.2 (6)	4.63 ± 0.85 (6)	4.18 ± 0.27 (5)	3.40 ± 0.73 (5)	4.99 ± 0.82 (6)
Gonadal WAT	104.9 ± 5.1 (5)	244.7 ± 116.3 (5)	95.6 ± 24.3 (5)	68.6 ± 11.7 (6)	2.3 ± 0.5 (6)	**3.5 ± 1.4# (5)**	2.9 ± 0.7 (4)	**10.5 ± 2.6# (6)**	519.7 ± 105.7 (6)	554.9 ± 82.8 (5)	583.6 ± 198.6 (5)	561.9 ± 105.0 (6)	3.19 ± 0.50 (5)	4.22 ± 0.83 (5)	3.13 ± 1.03 (5)	2.96 ± 0.58 (6)

Data are presented as means ± s.e.m., with sample sizes in parentheses. Bolded values indicate statistical significance. *Significant main population effect ($P < 0.05$). # Significant main acclimation effect ($P<0.05$). Statistics summary of F and P values for 2-way ANOVA for population, acclimation, and population × acclimation interaction is found in Table S5.

Journal of Experimental Biology • Supplementary information

Table S4. Central organ average mass (mg), specific tissue activity (^{14}C-counts per minute (CPM) per mg tissue), and total tissue activity (total tissue CPM per g body mass) of highland and lowland deer mice acclimated to thermoneutral conditions (TN; 30ºC, 21kPa O_2) or cold hypoxia (CH; 5ºC, 12kPa O_2) exposed to a maximal cold challenge in hypoxia (cold-induced $\dot{V}O_2$max).

Tissue	Tissue mass (mg)				Specific activity (CPM mg $^{-1}$)				Total activity (CPM g $^{-1}$ body weight)			
	Highlanders		Lowlanders		Highlanders		Lowlanders		Highlanders		Lowlanders	
	TN	CH	TN	CH	TN	CH	TN	CH	TN	CH	TN	CH
Liver	**676.0 ± 69.9* (6)**	**755.7 ± 38.6*# (5)**	469.2 ± 51.0 (5)	**692.6 ± 58.9# (6)**	15.6 ± 2.1 (6)	**10.6 ± 1.1# (5)**	25.7 ± 5.6 (5)	**11.7 ± 1.9# (6)**	104.2 ± 15.2 (6)	85.5 ± 17.7 (5)	87.6 ± 14.4 (5)	68.0 ± 3.5 (5)
Left Ventricle	72.6 ± 2.7 (6)	88.5 ± 2.7 (4)	52.9 ± 3.7 (5)	**104.8 ± 11.7‡ (6)**	66.8 ± 8.2 (6)	**34.0 ± 5.9# (5)**	82.4 ± 7.8 (5)	**28.7 ± 4.7# (6)**	5159.0 ± 609.2 (6)	3922.9 ± 509.2 (5)	4003.3 ± 434.1 (5)	3983.8 ± 418.1 (6)
Right Ventricle	23.3 ± 2.4 (6)	**35.9 ± 2.7# (5)**	24.6 ± 3.2 (5)	**37.7 ± 4.9# (6)**	206.1 ± 32.7 (6)	**92.3 ± 15.3# (5)**	186.6 ± 45.3 (5)	**78.5 ± 16.3# (6)**	1613.9 ± 278.7 (6)	1540.3 ± 264.4 (5)	1646.5 ± 128.8 (5)	1361.0 ± 139.7 (6)
Diaphragm	**50.0 ± 5.0* (6)**	**75.1 ± 5.4*# (5)**	46.6 ± 4.6 (5)	**55.2 ± 5.0# (6)**	59.9 ± 11.2 (6)	**32.2 ± 8.5# (5)**	60.0 ± 8.6 (5)	**38.6 ± 4.3# (6)**	2180.0 ± 356.5 (6)	2232.2 ± 392.9 (5)	2174.5 ± 317.2 (5)	1663.9 ± 250.6 (6)

Data are presented as means ± s.e.m., with sample sizes in parentheses. Values for liver *total activity* are ×10^3. Bolded values indicate statistical significance. *Significant main population effect (P <0.05). # Significant main acclimation effect (P<0.05). Significant differences in population × acclimation interactions result from Holm Sidak post hoc tests (P < 0.05). ‡Significantly different than thermoneutral conditions, within a population. Statistics summary of F and P values for 2-way ANOVA for population, acclimation, and population × acclimation interaction is found in Table S5.

Journal of Experimental Biology • Supplementary information

Table S5. Statistics summary of Tables S1, S2, S3 and S4. F and P values for 2-way ANOVA are shown. Population (pop), acclimation (Acc), and population × acclimation (Pop x Acc). Bolded values indicate statistical significance. CPM = ^{14}C-counts per minute.

	Tissue mass (mg)			Specific activity (CPM mg $^{-1}$)			Total activity (CPM g $^{-1}$ body weight)			Relative tissue uptake (%)		
Central/Core Muscles	Pop	Acc	Pop x Acc	Pop	Acc	Pop x Acc	Pop	Acc	Pop x Acc	Pop	Acc	Pop x Acc
Masseter	$F_{1,18}$ = 0.02<br>P = 0.89	**$F_{1,18}$ = 9.43<br>P < 0.01**	**$F_{1,18}$ = 4.55<br>P = 0.05**	$F_{1,17}$ = 1.31<br>P = 0.27	**$F_{1,17}$ = 4.53<br>P = 0.05**	**$F_{1,17}$ = 4.64<br>P = 0.05**	$F_{1,17}$ = 2.57<br>P = 0.13	$F_{1,17}$ = 1.60<br>P = 0.22	$F_{1,17}$ = 0.87<br>P = 0.37	$F_{1,17}$ = 0.94<br>P = 0.35	**$F_{1,17}$ = 4.73<br>P = 0.04**	$F_{1,17}$ = 0.81<br>P = 0.38
Erector Spinae	$F_{1,18}$ = 3.80<br>P = 0.07	$F_{1,18}$ = 0.10<br>P = 0.76	$F_{1,18}$ = 1.83<br>P = 0.19	$F_{1,18}$ = 0.57<br>P = 0.46	$F_{1,18}$ = 0.53<br>P = 0.47	$F_{1,18}$ = 2.99<br>P = 0.10	**$F_{1,17}$ = 15.75<br>P < 0.01**	**$F_{1,17}$ = 5.15<br>P = 0.04**	$F_{1,17}$ = 0.01<br>P = 0.91	$F_{1,18}$ = 0.45<br>P = 0.51	**$F_{1,18}$ = 4.45<br>P = 0.05**	$F_{1,18}$ = 0.35<br>P = 0.56
Trapezius	$F_{1,18}$ = 0.13<br>P = 0.73	$F_{1,18}$ = 0.38<br>P = 0.54	**$F_{1,18}$ = 7.35<br>P = 0.01**	$F_{1,18}$ = 1.08<br>P = 0.31	$F_{1,18}$ = 2.66<br>P = 0.12	$F_{1,18}$ = 3.60<br>P = 0.07	$F_{1,17}$ = 3.02<br>P = 0.10	$F_{1,17}$ = 0.14<br>P = 0.71	$F_{1,17}$ = 0.59<br>P = 0.45	$F_{1,18}$ = 2.29<br>P = 0.15	$F_{1,18}$ = 0.72<br>P = 0.41	$F_{1,18}$ = 4.13<br>P = 0.06
Gluteus	$F_{1,17}$ = 1.78<br>P = 0.20	$F_{1,17}$ = 3.07<br>P = 0.10	$F_{1,17}$ = 1.39<br>P = 0.26	$F_{1,18}$ = 2.13<br>P = 0.16	$F_{1,18}$ = 1.10<br>P = 0.31	$F_{1,18}$ = 2.72<br>P = 0.12	$F_{1,18}$ = 1.84<br>P = 0.19	$F_{1,18}$ = 0.72<br>P = 0.41	$F_{1,18}$ = 0.14<br>P = 0.71	$F_{1,18}$ = 1.57<br>P = 0.23	$F_{1,18}$ = 0.69<br>P = 0.42	$F_{1,18}$ = 0.02<br>P = 0.88
Upper Hindlimb Muscles												
Rectus Femoris	**$F_{1,18}$ = 4.45<br>P = 0.05**	$F_{1,18}$ = 0.07<br>P = 0.80	$F_{1,18}$ = 1.41<br>P = 0.25	$F_{1,18}$ = 0.93<br>P = 0.35	$F_{1,18}$ = 0.01<br>P = 0.91	$F_{1,18}$ = 2.69<br>P = 0.12	$F_{1,18}$ < 0.01<br>P = 0.94	$F_{1,18}$ = 1.35<br>P = 0.26	$F_{1,18}$ = 0.73<br>P = 0.40	**$F_{1,18}$ = 18.05<br>P < 0.01**	**$F_{1,18}$ = 4.74<br>P = 0.04**	$F_{1,18}$ = 0.27<br>P = 0.61
Vastus Lateralis	$F_{1,18}$ = 0.23<br>P = 0.64	$F_{1,18}$ = 0.34<br>P = 0.57	$F_{1,18}$ = 0.89<br>P = 0.36	$F_{1,17}$ = 0.40<br>P = 0.54	$F_{1,17}$ = 1.70<br>P = 0.21	$F_{1,17}$ = 0.76<br>P = 0.40	$F_{1,17}$ = 2.04<br>P = 0.17	$F_{1,17}$ = 2.92<br>P = 0.11	$F_{1,17}$ = 1.04<br>P = 0.32	$F_{1,18}$ = 0.78<br>P = 0.39	$F_{1,18}$ = 4.08<br>P = 0.06	$F_{1,18}$ = 0.01<br>P = 0.91
Vastus Medialis	$F_{1,18}$ < 0.01<br>P = 0.95	$F_{1,18}$ = 2.673<br>P = 0.12	$F_{1,18}$ < 0.01<br>P = 0.95	$F_{1,17}$ = 0.55<br>P = 0.47	$F_{1,17}$ = 2.53<br>P = 0.13	$F_{1,17}$ = 1.04<br>P = 0.32	$F_{1,17}$ = 2.84<br>P = 0.11	$F_{1,17}$ = 1.06<br>P = 0.32	$F_{1,17}$ = 1.07<br>P = 0.32	$F_{1,17}$ = 0.09<br>P = 0.76	$F_{1,17}$ = 2.66<br>P = 0.12	$F_{1,17}$ = 0.76<br>P = 0.40
Bicep Femoris	$F_{1,18}$ = 2.07<br>P = 0.17	$F_{1,18}$ = 1.92<br>P = 0.18	**$F_{1,18}$ = 6.91<br>P = 0.02**	$F_{1,18}$ = 1.56<br>P = 0.23	$F_{1,18}$ = 0.23<br>P = 0.64	$F_{1,18}$ = 3.57<br>P = 0.08	$F_{1,18}$ = 1.54<br>P = 0.23	$F_{1,18}$ = 2.273<br>P = 0.15	$F_{1,18}$ = 0.04<br>P = 0.85	$F_{1,18}$ = 2.92<br>P = 0.10	**$F_{1,18}$ = 8.32<br>P < 0.01**	$F_{1,18}$ = 2.02<br>P = 0.17
Semitendinosus	$F_{1,18}$ = 2.62<br>P = 0.12	$F_{1,18}$ = 0.31<br>P = 0.58	$F_{1,18}$ = 2.24<br>P = 0.15	$F_{1,17}$ = 0.77<br>P = 0.39	$F_{1,17}$ = 0.77<br>P = 0.39	$F_{1,17}$ = 2.29<br>P = 0.15	$F_{1,18}$ = 0.64<br>P = 0.44	$F_{1,18}$ = 1.97<br>P = 0.18	$F_{1,18}$ = 0.24<br>P = 0.63	$F_{1,18}$ = 1.90<br>P = 0.18	$F_{1,18}$ = 2.56<br>P = 0.13	$F_{1,18}$ = 0.01<br>P = 0.92
Lower Hindlimb Muscles												
Red Gastrocnemius	**$F_{1,17}$ = 7.15<br>P = 0.02**	$F_{1,17}$ < 0.01<br>P = 0.94	$F_{1,17}$ = 0.86<br>P = 0.37	$F_{1,18}$ = 2.33<br>P = 0.14	$F_{1,18}$ = 0.54<br>P = 0.47	$F_{1,18}$ = 0.28<br>P = 0.60	$F_{1,18}$ = 0.09<br>P = 0.77	$F_{1,18}$ = 0.32<br>P = 0.56	$F_{1,18}$ = 0.99<br>P = 0.33	**$F_{1,18}$ = 6.87<br>P = 0.02**	$F_{1,18}$ = 0.63<br>P = 0.44	$F_{1,18}$ = 0.01<br>P = 0.92
White Gastrocnemius	**$F_{1,17}$ = 4.86<br>P = 0.04**	$F_{1,17}$ = 1.27<br>P = 0.28	**$F_{1,17}$ = 4.82<br>P = 0.04**	$F_{1,18}$ = 1.96<br>P = 0.18	$F_{1,18}$ = 0.41<br>P = 0.53	**$F_{1,18}$ = 4.68<br>P = 0.04**	$F_{1,18}$ = 0.37<br>P = 0.55	**$F_{1,18}$ = 4.90<br>P = 0.04**	$F_{1,18}$ = 0.02<br>P = 0.89	$F_{1,18}$ = 3.37<br>P = 0.08	**$F_{1,18}$ = 7.17<br>P = 0.02**	$F_{1,18}$ = 0.52<br>P = 0.48
Soleus	$F_{1,16}$ = 3.43<br>P = 0.08	$F_{1,16}$ = 0.06<br>P = 0.80	$F_{1,16}$ = 2.31<br>P = 0.15	**$F_{1,16}$ = 4.73<br>P = 0.05**	$F_{1,16}$ = 0.44<br>P = 0.52	$F_{1,16}$ = 2.19<br>P = 0.16	$F_{1,16}$ = 0.65<br>P = 0.43	$F_{1,16}$ = 2.92<br>P = 0.11	$F_{1,16}$ = 0.80<br>P = 0.38	$F_{1,16}$ = 2.08<br>P = 0.17	$F_{1,16}$ = 2.47<br>P = 0.14	$F_{1,16}$ = 2.34<br>P = 0.15
Tibialis Anterior	$F_{1,17}$ = 0.80<br>P = 0.38	$F_{1,17}$ = 0.12<br>P = 0.73	$F_{1,17}$ = 1.87<br>P = 0.19	$F_{1,17}$ = 0.11<br>P = 0.74	$F_{1,17}$ = 0.40<br>P = 0.54	**$F_{1,17}$ = 5.38<br>P = 0.03**	$F_{1,17}$ < 0.01<br>P = 0.98	**$F_{1,17}$ = 4.80<br>P = 0.04**	$F_{1,17}$ = 0.82<br>P = 0.38	**$F_{1,17}$ = 12.40<br>P < 0.01**	**$F_{1,17}$ = 10.49<br>P < 0.01**	$F_{1,17}$ = 0.02<br>P = 0.88
Extensor Digitorum Longus	$F_{1,18}$ = 1.72<br>P = 0.21	$F_{1,18}$ = 3.26<br>P = 0.09	$F_{1,18}$ = 1.24<br>P = 0.28	$F_{1,18}$ = 0.85<br>P = 0.37	$F_{1,18}$ < 0.01<br>P >0.99	$F_{1,18}$ = 3.33<br>P = 0.09	$F_{1,18}$ = 0.21<br>P = 0.65	$F_{1,18}$ = 1.74<br>P = 0.20	$F_{1,18}$ = 0.21<br>P = 0.66	$F_{1,17}$ = 3.46<br>P = 0.08	$F_{1,17}$ = 2.37<br>P = 0.14	$F_{1,17}$ = 3.19<br>P = 0.09
Forelimb Muscles												
Bicep Brachii	**$F_{1,16}$ = 9.16<br>P < 0.01**	$F_{1,16}$ = 0.46<br>P = 0.51	$F_{1,16}$ = 1.75<br>P = 0.20	$F_{1,16}$ = 3.20<br>P = 0.09	$F_{1,16}$ = 1.45<br>P = 0.25	$F_{1,16}$ = 1.91<br>P = 0.19	$F_{1,16}$ = 1.88<br>P = 0.19	$F_{1,16}$ > 0.01<br>P = 0.95	$F_{1,16}$ = 0.03<br>P = 0.87	**$F_{1,16}$ = 8.57<br>P < 0.01**	$F_{1,16}$ = 0.09<br>P = 0.76	$F_{1,16}$ = 0.58<br>P = 0.46
Triceps	$F_{1,18}$ = 2.78<br>P = 0.11	$F_{1,18}$ = 0.42<br>P = 0.52	$F_{1,18}$ = 1.17<br>P = 0.29	$F_{1,18}$ = 1.45<br>P = 0.24	$F_{1,18}$ = 0.37<br>P = 0.55	$F_{1,18}$ = 2.58<br>P = 0.13	$F_{1,18}$ = 0.02<br>P = 0.88	$F_{1,18}$ = 0.42<br>P = 0.52	$F_{1,18}$ = 0.04<br>P = 0.85	**$F_{1,18}$ = 6.09<br>P = 0.02**	$F_{1,18}$ = 1.11<br>P = 0.31	$F_{1,18}$ = 0.34<br>P = 0.57

Journal of Experimental Biology • Supplementary information

Supplemental Table 1. Continued…												
	Tissue mass (mg)			Specific activity (CPM mg $^{-1}$)			Total activity (CPM g $^{-1}$ body weight)			Relative tissue uptake (%)		
	Pop	Acc	Pop x Acc	Pop	Acc	Pop x Acc	Pop	Acc	Pop x Acc	Pop	Acc	Pop x Acc
Brown Adipose Tissue (BAT)												
Auxiliary BAT	$F_{1,18}$ = 0.48, P = 0.50	$F_{1,18}$ = 1.53, P = 0.23	$F_{1,18}$ = 0.05, P = 0.83	$F_{1,16}$ = 1.66, P = 0.22	**$F_{1,16}$ = 8.25, P = 0.01**	$F_{1,16}$ = 1.49, P = 0.24	**$F_{1,18}$ = 5.07, P = 0.04**	$F_{1,18}$ = 1.15, P = 0.30	$F_{1,18}$ < 0.01, P = 0.99	$F_{1,17}$ = 1.40, P = 0.25	$F_{1,17}$ = 2.10, P = 0.17	$F_{1,17}$ = 0.53, P = 0.48
Interscapular BAT	$F_{1,18}$ = 1.04, P = 0.32	$F_{1,18}$ = 1.04, P = 0.32	$F_{1,18}$ = 0.68, P = 0.42	$F_{1,17}$ < 0.01, P > 0.99	**$F_{1,17}$ = 7.22, P = 0.02**	$F_{1,17}$ = 0.02, P = 0.90	**$F_{1,18}$ = 12.87, P < 0.01**	$F_{1,18}$ = 2.66, P = 0.12	$F_{1,18}$ = 0.10, P = 0.75	**$F_{1,18}$ = 9.14, P < 0.01**	**$F_{1,18}$ = 8.66, P < 0.01**	$F_{1,18}$ = 2.03, P = 0.17
White Adipose Tissue (WAT)												
Inguinal WAT	$F_{1,16}$ = 3.04, P = 0.10	$F_{1,16}$ = 0.90, P = 0.36	$F_{1,16}$ = 0.66, P = 0.43	$F_{1,16}$ = 2.92, P = 0.11	$F_{1,16}$ = 2.33, P = 0.15	$F_{1,16}$ = 3.86, P = 0.07	$F_{1,18}$ = 2.74, P = 0.12	$F_{1,18}$ = 1.16, P = 0.30	$F_{1,18}$ = 1.32, P = 0.27	$F_{1,18}$ = 0.08, P = 0.78	$F_{1,18}$ = 0.58, P = 0.46	$F_{1,18}$ = 1.89, P = 0.19
Gonadal	$F_{1,17}$ = 2.66, P = 0.12	$F_{1,17}$ = 0.98, P = 0.34	$F_{1,17}$ = 2.15, P = 0.16	**$F_{1,17}$ = 4.89, P = 0.04**	**$F_{1,17}$ = 6.39, P = 0.02**	$F_{1,17}$ = 3.39, P = 0.08	$F_{1,18}$ = 0.08, P = 0.78	$F_{1,18}$ < 0.01, P = 0.96	$F_{1,18}$ = 0.05, P = 0.83	$F_{1,17}$ = 0.78, P = 0.39	$F_{1,17}$ = 0.33, P = 0.57	$F_{1,17}$ = 0.65, P = 0.43
Central Organs												
Liver	**$F_{1,18}$ = 5.38, P = 0.03**	**$F_{1,18}$ = 6.79, P = 0.02**	$F_{1,18}$ = 1.53, P = 0.23	$F_{1,18}$ = 3.47, P = 0.08	**$F_{1,18}$ = 9.89, P < 0.01**	$F_{1,18}$ = 2.19, P = 0.16	$F_{1,17}$ = 1.48, P = 0.24	$F_{1,17}$ = 1.87, P = 0.19	$F_{1,17}$ < 0.01, P = 0.97			
Left Ventricle	$F_{1,17}$ = 0.05, P = 0.82	**$F_{1,17}$ = 21.14, P < 0.01**	**$F_{1,17}$ = 5.99, P = 0.03**	$F_{1,18}$ = 0.56, P = 0.46	**$F_{1,18}$ = 40.06, P < 0.01**	$F_{1,18}$ = 2.34, P = 0.14	$F_{1,18}$ = 1.16, P = 0.30	$F_{1,18}$ = 1.53, P = 0.23	$F_{1,18}$ = 1.43, P = 0.25			
Right Ventricle	$F_{1,18}$ = 0.19, P = 0.67	**$F_{1,18}$ = 13.03, P < 0.01**	$F_{1,18}$ < 0.01, P = 0.94	$F_{1,18}$ = 0.32, P = 0.58	**$F_{1,18}$ = 14.07, P < 0.01**	$F_{1,18}$ < 0.01, P = 0.92	$F_{1,18}$ = 0.11, P = 0.74	$F_{1,18}$ = 0.68, P = 0.42	$F_{1,18}$ = 0.24, P = 0.63			
Diaphragm	**$F_{1,18}$ = 5.37, P = 0.03**	**$F_{1,18}$ = 11.21, P < 0.01**	$F_{1,18}$ = 2.71, P = 0.12	$F_{1,18}$ = 0.14, P = 0.71	**$F_{1,18}$ = 8.19, P = 0.01**	$F_{1,18}$ = 0.13, P = 0.72	$F_{1,18}$ = 0.75, P = 0.40	$F_{1,18}$ = 0.48, P = 0.50	$F_{1,18}$ = 0.72, P = 0.41			

Journal of Experimental Biology • Supplementary information
